# Complete versus culprit-only revascularization in patients with ST-segment elevation myocardial infarction and multivessel disease: a meta-analysis of randomized trials

**DOI:** 10.1186/s12872-019-1073-8

**Published:** 2019-04-22

**Authors:** Haiyan Xu, Xiwen Zhang, Jiangjin Li, Hailang Liu, Xiao Hu, Jing Yang

**Affiliations:** Department of Cardiology, The affiliated Huaian No.1 People’s Hospital of Nanjing Medical University, 6 Beijing Road West, Huai’an, 223300 Jiangsu China

**Keywords:** Complete revascularization, Infarct-related artery only revascularization, ST-elevation myocardial infarction, Multivessel disease

## Abstract

**Background:**

The best strategy for the treatment of the non-infarct artery in patients with ST-elevation myocardial infarction (STEMI) and multivessel disease (MVD) undergoing primary percutaneous coronary intervention (PCI) is not yet defined.

**Methods:**

We searched the literature for randomized controlled trials (RCTs) that compared complete revascularization (CR) with infarct-related coronary artery (IRA) only revascularization in hemodynamically stable patients with STEMI. Random effect risk ratios (RRs) were calculated for clinical outcomes.

**Results:**

Nine RCTs with 2989 patients were included. No significant difference in all-cause mortality emerged between CR and IRA-only groups (relative risk [RR] = 0.74; 95% confidence interval [CI]: 0.52 to 1.04; *p* = 0.08). Compared with IRA-only, CR was associated with significantly lower rates of major adverse cardiac events (MACE) (RR = 0.53; 95% CI: 0.41 to 0.68; *p* < 0.001), cardiac death (RR = 0.48; 95% CI: 0.29 to 0.79; *p* = 0.004) and repeat revascularization (RR = 0.38; 95% CI: 0.30 to 0.47; *p* < 0.001). In subgroups analysis, immediate complete revascularization (ICR) reduced the risk of all-cause mortality (RR = 0.62; 95% CI: 0.39 to 0.97; *p* = 0.04), whereas staged complete revascularization (SCR) did not show any significant benefit in all-cause mortality (RR = 0.92; 95% CI: 0.46 to 1.86; *p* = 0.82). Stroke, contrast-induced nephropathy and major bleeding were not different between CR and IRA-only.

**Conclusions:**

For patients with STEMI and multivessel disease undergoing primary PCI, complete revascularization did not decrease the risk of all-cause mortality in current evidence from randomized trials. When feasible, immediate complete revascularization might be considered in patients with STEMI and multivessel disease.

**Electronic supplementary material:**

The online version of this article (10.1186/s12872-019-1073-8) contains supplementary material, which is available to authorized users.

## Background

Patients with acute ST-segment elevation myocardial infarction (STEMI) are effectively treated with primary percutaneous coronary intervention (PCI) of the infarct-related coronary artery. Approximately, 40–60% of these patients have multivessel disease (MVD) and are associated with worse clinical outcomes compared with those have single-vessel disease [1–3]. The American College of Cardiology/American Heart Association have updated their guidelines recommendation from III to IIb for primary PCI of the non-infarct-related coronary artery in hemodynamically stable patients with STEMI and MVD [4]. Current European Society of Cardiology guidelines support intervention of the non-IRA at the time of primary PCI (Class IIa indication) [5].

Most recent randomized controlled trials (RCTs) reported that complete revascularization (CR) for hemodynamically stable patients with STEMI and MVD at the time of primary PCI might have beneficial effects [6, 7]. However, these trials are limited by sample sizes and not powered to detect differences in all-cause mortality or myocardial infarction (MI). Moreover, the optimal strategy of complete revascularization, either immediate complete revascularization (ICR) during primary PCI or staged complete revascularization (SCR), and its impact on mortality is still unclear. Therefore, we conducted this meta-analysis of RCTs to assess whether complete revascularization can reduce all-cause mortality in patients with STEMI and MVD and to determine the possible strategy of complete revascularization.

## Methods

### Data sources

**Fig. 1:**
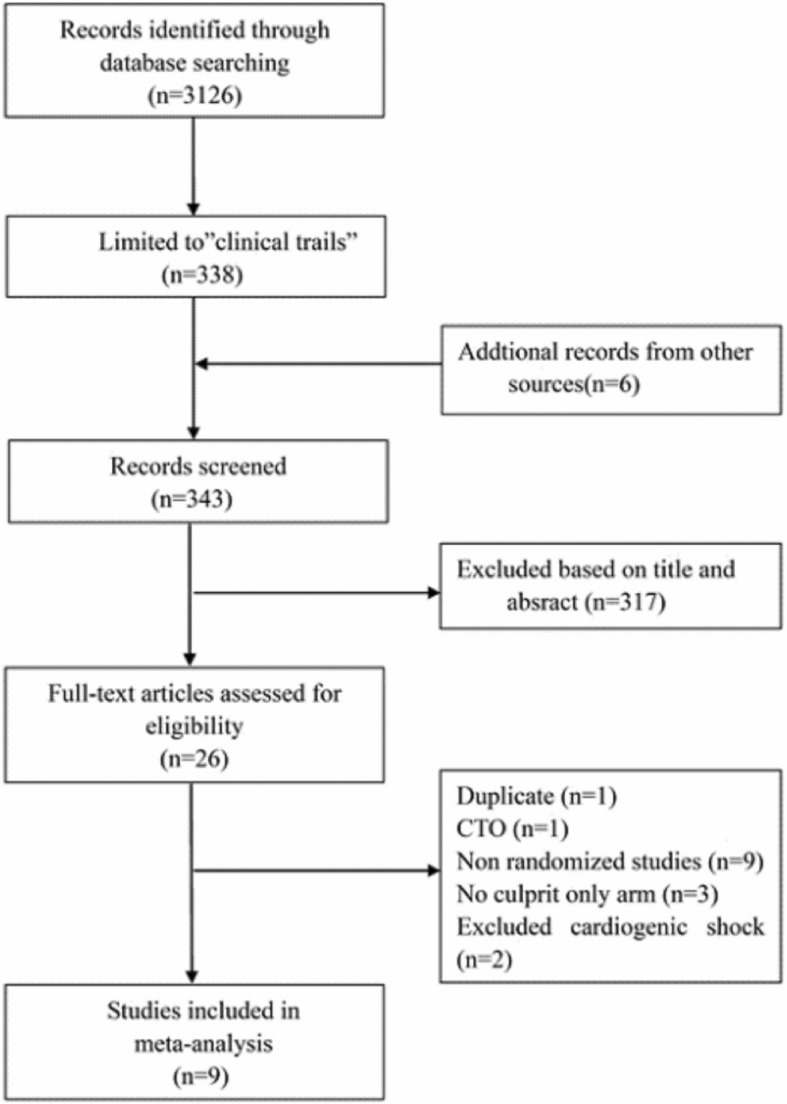
Flow diagram showing selection of studies for final analysis

We searched PubMed, MEDLINE, EMBASE, the Cochrane Central Register of Controlled Trials (CENTRAL), and Clinical Trials.gov for RCTs using the terms “myocardial infarction”, “percutaneous coronary intervention”, “coronary angioplasty” and “multivessel”. All RCTs published until 1 August 2018 were identified. The reference lists of the retrieved articles were manually searched for additional potential articles. The search algorithm is shown in Fig. 1. The meta-analysis was performed according to the Preferred Reporting Items for Systematic Reviews and Meta-Analyses (PRISMA) statement [8].

### Selection criteria

The inclusion criteria were:1) studies of hemodynamically stable patients with STEMI and MVD at the time of primary PCI; 2) randomized clinical trials comparing complete versus IRA-only revascularization; 3) main outcomes of interest including (mortality, MI, and revascularization) reported. Trials including chronic total occlusion were excluded.

### Data extraction and quality assessment

Two authors (JJ.L and HL.L) independently assessed trial eligibility and extracted data. Any disagreements were resolved by consensus of the authors. We performed objective assessment of the trials using a standardized data collection form. The data extracted from the trials included the year of publication, sample size, duration of follow-up, definitions of endpoints used in each study, clinical outcomes (including major adverse cardiac events [MACE], all-cause mortality, MI, repeat revascularization, stoke, contrast-induced nephropathy [CIN], and major bleeding). The trial bias risk was assessed by Cochrane Collaboration guidelines [9].

### Outcomes measures

The primary outcome was all-cause mortality. Secondary outcomes included composite MACE, cardiac death, nonfatal MI, repeat revascularization, stroke, CIN and major bleeding.

### Statistical analysis

The meta-analysis was performed using STATA software version 12 (STATA Corporation; College Station, Texas). We analyzed outcomes in an intention-to-treat analysis. Risk ratios (RRs) and 95% confidence intervals (CIs) were used as summary statistics. Fixed-effect model of Mantel-Haenszel was used to assess the overall estimate; however, a random-effect model was chosen to calculate pooled RRs when heterogeneity existed. Heterogeneity was assessed by I^2^ test and its *P* value. I^2^ < 25% was defined as low heterogeneity and > 75% was defined as significant heterogeneity. Pre-specified subgroup analysis was performed according to strategy of complete revascularization (predominantly immediate revascularization or staged revascularization). Serially leave-one-out analysis was used to eliminate sources of heterogeneity in sensitivity analysis. Publication bias was visually assessed using funnel plots. A 2-tailed *P* < 0.05 was considered statistically significant.

## Results

### Characteristics of included studies

Our search identified 3126 articles, among which 343 abstracts were screened. Nine randomized studies [6, 7, 10–16] enrolling 2989 patients met the inclusion criteria and were included in this meta-analysis. The characteristics of the included studies are presented in Table 1. A total of 1582 patients underwent IRA-only revascularization. Among the 1407 patients who underwent complete revascularization, 843 patients were assigned to the ICR group and 564 patients were assigned to the SCR group. In the study by Politi et al. [15]**,** 65 patients who underwent immediate complete revascularization during primary PCI were included in the ICR group, whereas the 65 patients who underwent staged complete revascularization were included in SCR group. The timing of staged intervention was from 2 days up to 57 days in the SCR group. In Politi’s study [15], the time of patients who underwent staged complete revascularization of the non-infarct-related artery was 56.8 ± 12.9 days after primary PCI. All studies, except for Ghani [14], excluded patients with cardiogenic shock. Most of the studies employed angiography to estimate stenosis in the non-infarct artery. However, fractional flow reserve (FFR) was used to guide PCI for non-infarct-related coronary artery lesions in 3 studies [6, 7, 14]. The duration of the follow-up ranged from 6 to 38 months (23.0 months). Table 2 summarizes baseline characteristics of the patients.

**Table 1:** Characteristics of included studies

Study	Multivessel disease definition	MACE definition	Definition of complete revascularization	Timing of staged complete revascularization	Follow-up (months)
COMPARE-ACUTE 2017 [7]	IRA plus non-IRA or their major side branches of at least 2.0 mm diameter show ≥50% stenosis by QCA or visual assessment	All-causemortality, nonfatal MI, any revascularization, cerebrovascular events	Non-IRAs with at least 50% stenosis and who had a FFR ≤0.80 were revascularized with Everolimus DES	Within 3 days	12
Hamza et al. 2016 [10]	IRA plus at least 80% stenosis in non-IRA	Composite of all- cause mortality, recurrent MI, ischemia driven revascularization with PCI or CABG	N-IRAs with at least 80% stenosis were revascularized.	Within 3 days	6
DANAMI-3- PRIMULTI 2015 [6]	IRA plus > 50% stenosis in one or more non-IRA	Composite of all- cause mortality, reinfarction, or ischemia driven revascularization of non–IRA	Non-IRAs which were ≥ 2 mm in diameter with at least 50% stenosis and FFR < 0.8 or those with visually Estimated stenosis> 90% stenosis were treated with everolimus DES.	Within 2 days	27
PRAGUE − 132,015 [11]	at least 1 stenosis of non-IRA > 70% with diameter > 2.5 mm	All cause mortality, non- fatal MI and stroke.	NA	3–40 days	38
CvLPRIT 2015 [12]	IRA plus at least one non-IRA with at least one lesion> 70% single view/50% in two views	All-cause mortality, MI,HF, ischemia driven PCI OR CABG	Non-IRAs with at least > 70% stenosis in one view or > 50% stenosis in two views were revascularized with DES	Within 3 days	12
PRAMI 2013 [13]	IRA plus one or more non-IRA > 50% stenosis	Composite of death from cardiac cause, nonfatal MI, refractory angina.	Non-IRA stenoses > 50% were intervened	At the same procedure	23
Ghani et al. 2012 [14]	One or more stenoses of ≥50% (in at least one view visually or by QCA) in at least two major epicardial coronary arteries	Death, nonfatal reinfarction, unplanned revascularization	Vessel with significant stenosis vascularized if FFR < 0.75. For severe stenosis (> 90%) PCI performed without preceding FFR.	Within 3 weeks after STEMI	36
Politi et al. 2010 [15]	> 70% stenosis of at least two epicardial coronary arteries or their major branches	Death, reinfarction, rehospitalization for ACS and repeat coronary revascularization	Non-IRAs with PCI and angiographic residual stenosis of < 30% or TIMI flow grade of 3	56.8 ± 12.9 days	30
HELP AMI 2004 [16]	IRA plus at least 1–3 lesions in major non-IRA	Death, repeat MI, urgent revascularization	All suitable non-IRAs with heparin coated Bx velocity stents. Balloon dilatation alone was performed for lesions in vessels with diameter < 2.5 mm provided at least one non-IRA was treated with stents.	At the same procedure	12

*IRA* infarct-related artery only, *QCA* quantitative coronary angiography, *MACE* major adverse cardiac events, *MI* myocardial infarction, *ACS* acute coronary sydrome, *PCI* percutaneouscoronary intervention, *CABG* coronary artery bypass grafting, *FFR* fractional flow reserve, *TIMT* thrombolysis in myocardial infarction

**Table 2:** Baseline patient characteristics

Study	Number (CR/IRA-only) (n)	Male (CR/IRA-only) (%)	Age (CR/IRA-only) (years)	Hypertension (CR/IRA-only) (%)	Diabetes (CR/IRA-only) (%)	Previous MI (CR/IRA-only) (%)	Smoking (CR/IRA-only) (%)
COMPARE-ACUTE 2017 [7]	295/590	79/76	62/61	46/48	15/16	7.5/8.1	41/49
Hamza et al. 2016 [10]	50/50	82/86	56/52	26/36	100/100	10/6	72/78
DANAMI-3- PRIMULTI 2015 [6]	314/313	80/81	64/63	41/47	9/13	5/9	51/48
PRAGUE − 132,015 [11]	106/108	NA	NA	NA	NA	NA	NA
CvLPRIT 2015 [12]	150/146	85/77	64/65	37/36	13/14	4.8/3.6	34/37
PRAMI 2013 [13]	234/231	76/81	62/62	40/40	15/21	8/7	50/45
Ghani et al. 2012	79/40	80/81	62/61	26/43	6.3/5.0	6.3/4.9	44/48
Politi et al. 2010 [14]	130/84	77/78	65/67	57/60	23/24	NA	NA
HELP AMI 2004 [15]	52/17	88/85	64/65	37/59	12/41	NA	67/81
Study	Anterior MI (CR/IRA-only) (%)	Procedure time (CR/IRA-only) (min)	Contrast Media (CR/IRA-only) (ml)	Glycoprotein IIb/IIIa Inhibitors (CR/IRA-only) (%)	DES (CR/IRA-only) (years)		
COMPARE-ACUTE 2017 [7]	36/35	65/59	224/202	22/25	95/96		
Hamza et al. 2016 [10]	48/46	NA	NA	38/34	100/100		
DANAMI-3- PRIMULTI 2015 [6]	33/36	76/42	280/170	20/23	95/93		
PRAGUE − 132,015 [11]	NA	NA	NA	NA	NA		
CvLPRIT 2015 [12]	36/36	55/41	250/190	32/32	96/91		
PRAMI 2013 [13]	29/39	63/45	300/200	79/78	63/58		
Ghani et al. 2012 [14]	NA	NA	NA	45/46	23/17		
Politi et al. 2010 [15]	48/42	NA	NA	100/100	NA		
HELP AMI 2004 [16]	52/59	53/69	341/242	75/82	0/0		

*CR* complete revascularization, *IRA* infarct-related artery only, *DES* drug-eluting stent, *NA* not available

### All-cause mortality

**Fig. 2:**
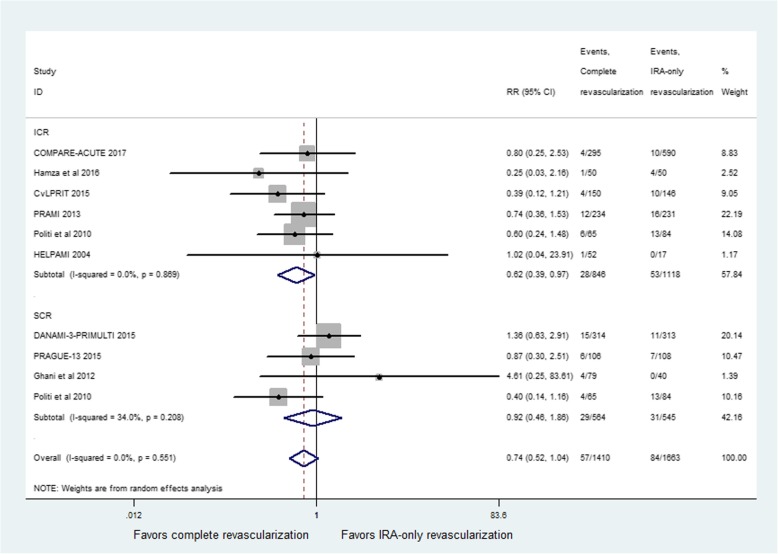
Relative risk for all-cause mortality for complete revascularization (CR) versus infarct-related coronary artery (IRA) only revascularization

All studies reported all-cause mortality. The incidence of all-cause mortality did not show a significant difference between CR and IRA-only groups (RR = 0.74; 95% CI: 0.52 to 1.04; *p* = 0.08, Fig. 2), with no heterogeneity among studies (I^2^ = 0%, *p* = 0.55). Compared with IRA-only revascularization, ICR significantly reduced the risk for all-cause mortality (RR = 0.62; 95% CI: 0.39 to 0.97; *p* = 0.04, Fig. 2). No heterogeneity was seen for the results (I^2^ = 0%, *p* = 0.87). However, no significant difference was found between SCR and IRA-only groups (RR = 0.92; 95% CI: 0.46 to 1.86; *p* = 0.82, Fig. 2), with modest heterogeneity of the results (I^2^ = 34%, *p* = 0.21). In the sensitivity analysis, exclusion of the study by Politi et al. resulted in a reduction of the heterogeneity to 0% with no impact on the result in the SCR group (RR = 1.29, 95% CI: 0.71–2.35; *p* = 0.40).

### Major adverse cardiac events

**Fig. 3:**
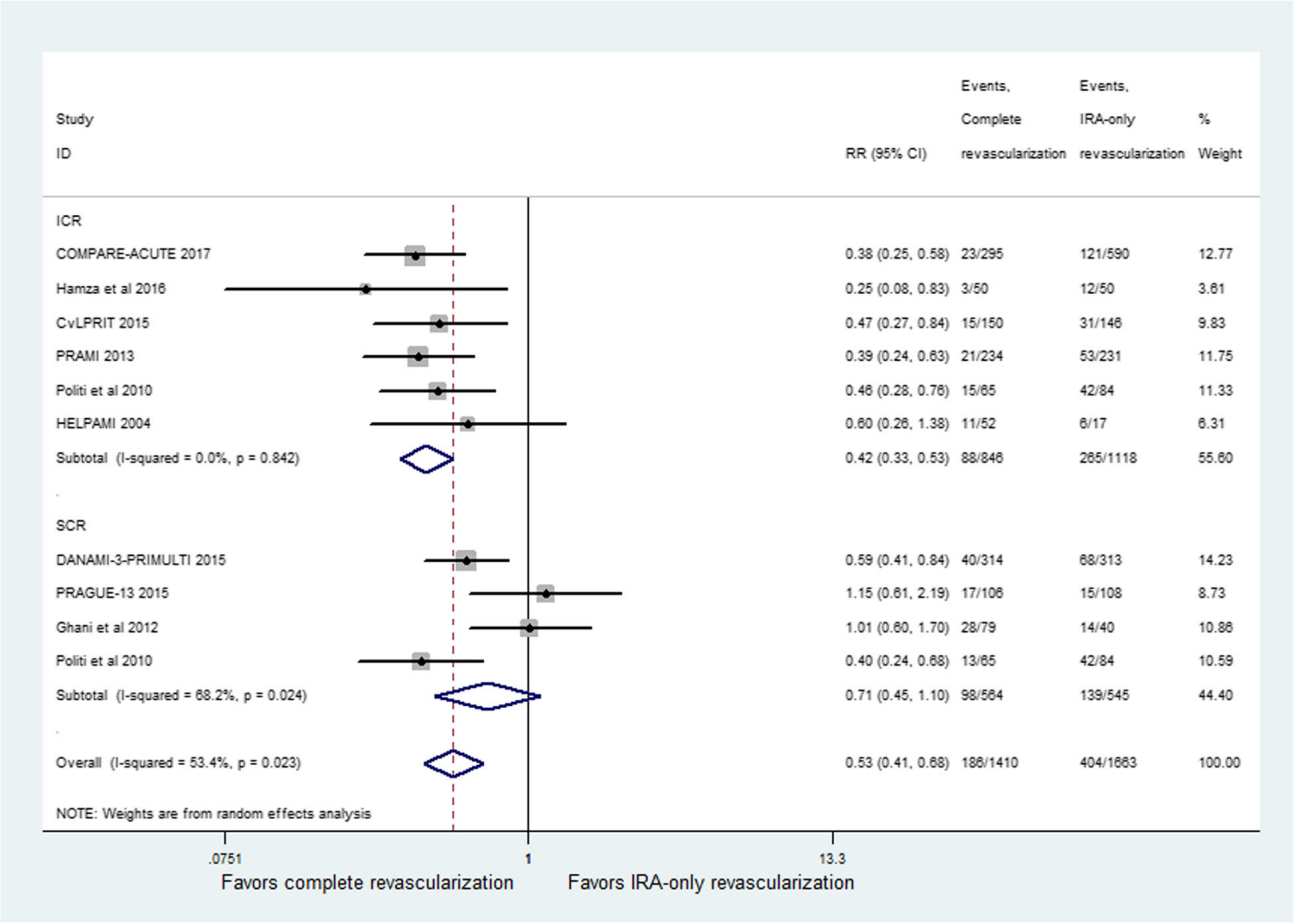
Relative risk for major adverse cardiac events (MACE) for complete revascularization (CR) versus infarct-related coronary artery (IRA) only revascularization

MACE was reported in all studies but its definition differed across all studies. The incidence of MACE showed a significant difference between CR and IRA-only groups (RR = 0.53; 95% CI: 0.41 to 0.68; *p* < 0.001, Fig. 3), with moderate heterogeneity among studies (I^2^ = 53.4%, *p* = 0.02). Compared with IRA-only revascularization, ICR significantly reduced the risk for MACE (RR = 0.42; 95% CI: 0.33 to 0.53; *p* < 0.001, Fig. 3). No heterogeneity was seen for the results (I^2^ = 0%, *p* = 0.84). The studies performing staged revascularization did not report any beneficial effect (RR = 0.71; 95% CI: 0.45 to 1.10; *p* = 0.12, Fig. 3), with high heterogeneity among studies (I^2^ = 68.2%, p = 0.02). The pooled results were not influenced by exclusion of PRAGUE-13Trial [11] and the study by Ghani et al. [14], with no evidence of heterogeneity among the CR group.

### Cardiac death

**Fig. 4:**
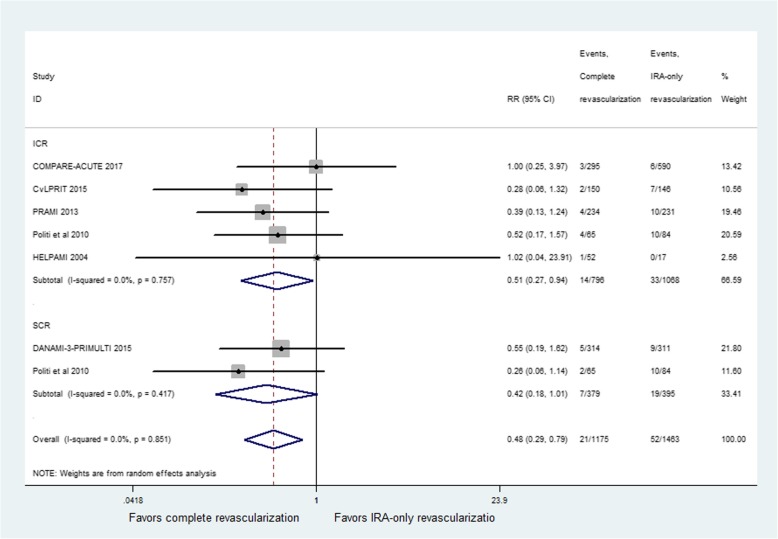
Relative risk for cardiac death for complete revascularization (CR) versus infarct-related coronary artery (IRA) only revascularization

Cardiac death was reported in six studies. The incidence of cardiac death showed a significant difference between CR and IRA-only groups (RR = 0.48; 95% CI: 0.29 to 0.79; *p* = 0.004, Fig. 4), with no heterogeneity among studies (I^2^ = 0%, *p* = 0.85). The similar result was obtained in ICR group (RR = 0.51; 95% CI: 0.27 to 0.94; *p* = 0.03) with no signs of heterogeneity (I^2^ = 0%, *p* = 0.76). However, the studies performing staged revascularization during primary PCI had a trend toward a reduction in cardiac death (RR = 0.42; 95% CI: 0.18 to 1.01; *p* = 0.054, Fig. 4) and no evidence of heterogeneity among studies (I^2^ = 0%, *p* = 0.42).

### Recurrent myocardial infarction

**Fig. 5:**
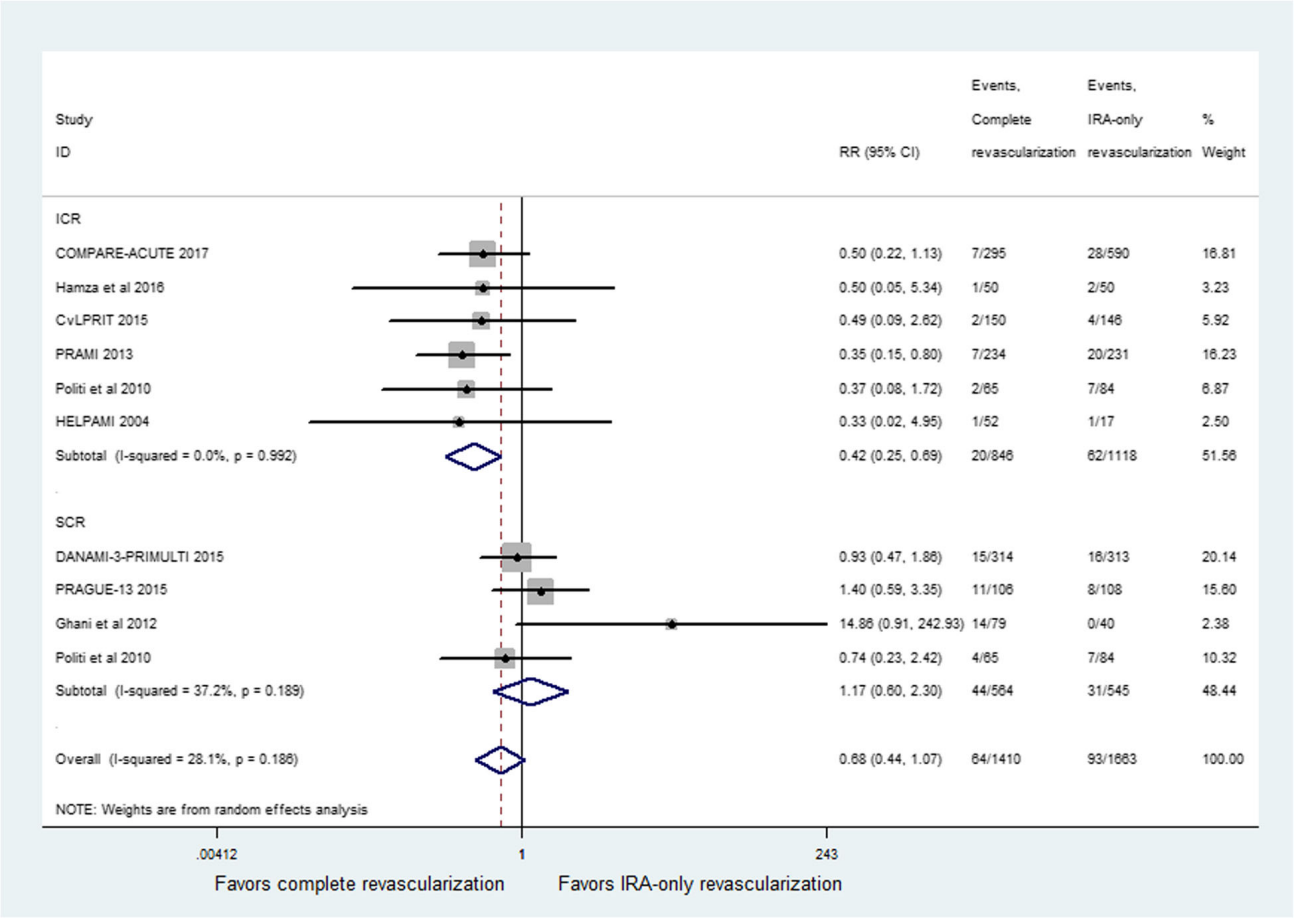
Relative risk for recurrent myocardial infarction(MI) for complete revascularization (CR) versus infarct-related coronary artery (IRA) only revascularization

Recurrent myocardial infarction was reported in all studies. The incidence of recurrent MI did not show a significant difference between CR and IRA-only groups (RR = 0.68; 95% CI: 0.44 to 1.07; *p* = 0.09, Fig. 5), with moderate heterogeneity among studies (I^2^ = 28.1%, *p* = 0.19). However, the studies performing immediate revascularization during primary PCI had a significant reduction in recurrent MI (RR = 0.42; 95% CI: 0.25 to 0.69; *p* = 0.001, Fig. 5) and no evidence of heterogeneity among studies (I^2^ = 0%, *p* = 0.99). The SCR group did not report any beneficial effect (RR = 1.17; 95% CI: 0.60 to 2.30; *p* = 0.64), with moderate heterogeneity among studies (I^2^ = 37.2%, *p* = 0.19). Exclusion of study by Ghani et al. [14] resulted in the heterogeneity of 0%. However, there was a significant difference between CR and IRA-only groups (RR = 0.66; 95% CI: 0.46 to 0.94; *p* = 0.02).

### Repeat revascularization

**Fig. 6:**
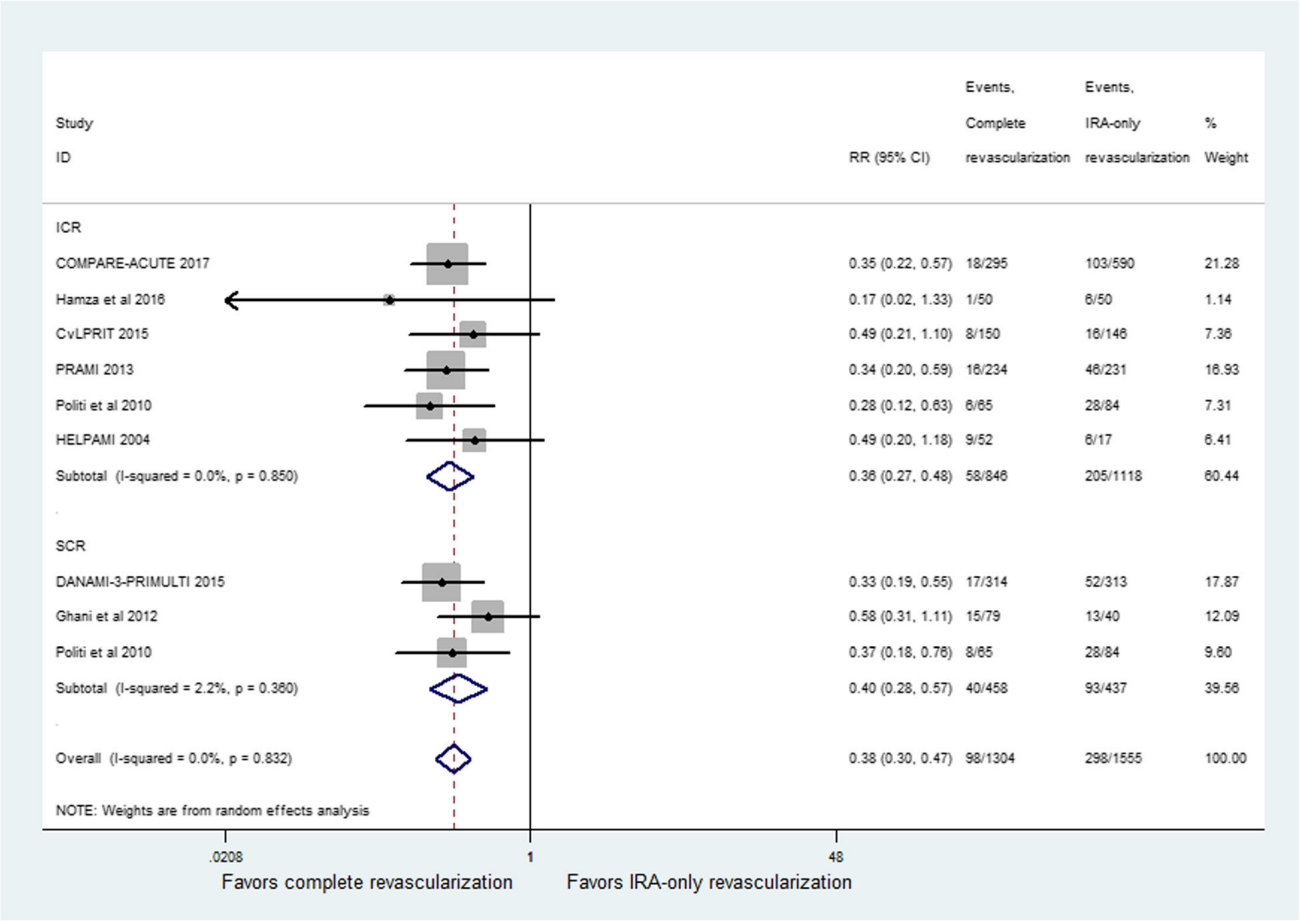
Relative risk for repeat revascularization for complete revascularization (CR) versus infarct-related coronary artery (IRA) only revascularization

Repeat revascularization was reported in eight studies. The incidence of repeat revascularization showed a significant difference between CR and IRA-only groups (RR = 0.38; 95% CI: 0.30 to 0.47; *p* < 0.001, Fig. 6), with no heterogeneity among studies (I^2^ = 0%, *p* = 0.83). The ICR group (RR = 0.36; 95% CI: 0.27 to 0.48; *p* < 0.001) and the SCR group (RR = 0.40; 95% CI: 0.28 to 0.57; *p* < 0.001) had similar results.

### Stroke

Stroke was reported in five studies. The incidence of stroke did not show a significant difference between CR and IRA-only groups (RR = 0.73; 95% CI: 0.22 to 2.39; *p* = 0.60, Additional file 1: Figure S1), with very low heterogeneity among studies (I^2^ = 9.9%, *p* = 0.35). The ICR group (RR = 0.54; 95% CI: 0.13 to 2.30; *p* = 0.41) and the SCR group (RR = 0.89; 95% CI: 0.03 to 23.04; *p* = 0.94) reported similar results.

### Contrast-induced nephropathy

Contrast-induced nephropathy was reported in five studies. The incidence of CIN did not show a significant difference between CR and IRA-only groups (RR = 0.84; 95% CI: 0.42 to 1.69; *p* = 0.63, Additional file 2: Figure S2), with no heterogeneity among studies (I^2^ = 0%, *p* = 0.81). Again, the ICR group (RR = 0.82; 95% CI: 0.28 to 2.41; *p* = 0.72) and the SCR group (RR = 0.86; 95% CI: 0.34 to 2.15; *p* = 0.74) had similar results.

### Major bleeding

Major bleeding was reported in four studies. The overall incidence of major bleeding was very low (1.4% vs. 1.9%), with no difference between CR and IRA-only groups (RR = 0.56; 95% CI: 0.24 to 1.27; *p* = 0.17, Additional file 3: Figure S3).

### Subgroup analysis

Trials using angiography had a significant risk reduction in risk for all-cause mortality (RR = 0.59; 95% CI: 0.39 to 0.89; *p* = 0.01), whereas trials using FFR did not report any beneficial effect (RR = 1.23; 95% CI: 0.66 to 2.29; *p* = 0.51, Additional file 4: Figure S4).

The follow-up time of 5 trails [6, 11, 13–15] was more than 12 months, whereas other 4 trails [7, 10, 12, 16] was not. There was a trend toward a reduction in all-cause mortality with complete revascularization in trails that the follow-up time was not more than 12 months (RR = 0.52; 95% CI: 0.25 to 1.09; *p* = 0.09, Additional file 5: Figure S5). The incidence of all-cause mortality did not show a significant difference between CR and IRA-only groups in trails that the follow-up time was more than 12 months (RR = 0.81; 95% CI: 0.54 to 1.21; *p* = 0.30, Additional file 5: Figure S5).

### Publication bias

Funnel plots did not suggest publication bias for any of the clinical outcomes (*p* = 1.00 for all-cause mortality, Additional file 6: Figure S6).

## Discussion

In this meta-analysis of nine randomized trials including 2989 patients with STEMI and MVD, we demonstrated that the risk of all-cause mortality was not different between complete revascularization and infarct-related coronary artery only revascularization. The present analysis found that a trend towards decrease in all-cause mortality in CR when compared with IRA-only, however it did not reach statistical significance. Immediate complete revascularization significantly reduced the risk for all-cause mortality compared with IRA-only; whereas staged revascularization did not show any benefit on the outcome.

Most data of observational studies had suggested that complete revascularization for multivessel disease at the time of primary PCI might be harmful [17–19]. Recent RCTs and meta-analyses showed that CR reduced the risk of a composite cardiovascular outcome as compared with IRA-only [6, 7, 20]. However, none of the RCTs demonstrated a significant difference between complete revascularization and IRA-only revascularization in death or MI, but only in MACE. In our analysis, there was no significant difference in all-cause mortality between CR and IRA-only among patients with STEMI and MVD undergoing primary PCI.

Subgroup analyses demonstrated a significant benefit of ICR compared with IRA-only. The risk of MACE, cardiac death, recurrent MI, and repeat revascularization were also significantly lower among those underwent ICR. The findings were consistent with a more recent network meta-analysis [21]. The timing of staged intervention was heterogeneous across the included trials. In this study by Politi and colleagues [15], the time of staged complete revascularization PCI of the non-IRA after primary PCI was 56.8 ± 12.9 days, which was longer than other trails**.** After exclusion of the study by Politi et al. [15], the results for all-cause mortality did not change. However, staged revascularization did not seem to be associated with any beneficial effect. Our results were consistent with other meta-analyses that suggested that immediate complete revascularization was associated with significant reduction in all-cause mortality [20–22]. Only one small trial [15] that compared immediate versus staged revascularization in STEMI patients with MVD, so it is not possible yet to directly compare these two strategies in this meta-analysis. An earlier network meta-analysis [23] reported the risk of all-cause mortality is not different between the two revascularization strategies. However, two more recent network meta-analyses [21, 22] that demonstrated the benefit of ICR in reducing all-cause mortality compared to SCR. The optimal revascularization strategy (CR versus IRA-only) in hemodynamically stable patients with STEMI and MVD continues to be debated. Therefore, large randomized trials might be required to clarify the benefit of immediate complete revascularization in STEMI patients with MVD.

The mechanisms by which immediate complete revascularization may improve prognosis in STEMI patients with MVD are probably related to pathophysiology of MI. First, ICR may theoretically decrease infarct size [24] and preserve left ventricular function [25]. Achieving complete revascularization as soon as possible may help reduce the risk for death and MI. Second, unstable plaques in the non-culprit lesion are associated with increased risk for acute coronary events. Hong et al. [26] reported that non-infarct-related artery plaque ruptures occurred in 17% of patients using intravascular ultrasound (IVUS) examination. Besides, there are several advantages of performing ICR, such as decreased risk of vascular complications and lower costs.

The meta results showed that the risk of MACE was significantly lower among those in the CR group, which was consistent with the prior meta-analyses [22, 23, 27]. This benefit was derived from the primarily reduced rate of repeat revascularization. Additionally, the incidence of cardiac death was lower with ICR than with IRA-only.

It has been reported that adding FFR measurements to assess ischemia during coronary angiography might reduce cardiac events in patients with stable coronary artery disease [28, 29]. However, whether the FFR measurements benefit in patients with STEMI is not clear. In this subgroup analysis of using FFR measurements, FFR-guided CR did not report any beneficial effect (RR = 1.23; 95% CI: 0.66 to 2.29; *p* = 0.51) compared to IRA-only. Both the DANAMI 3-PRIMULTI trial and COMPARE-ACUTE trail found that no differences in death between FFR-guided staged PCI and IRA-only. It may be that the disturbed microvascular function in the non-IRA territory in the early stage of STEMI affects the reliability of the technique.

Our results indicated that all-cause mortality had a trend toward a reduction in trails performing complete revascularization which the follow-up time were not more than 12 months. The exact mechanisms linking complete revascularization with better mortality maybe elucidated by less rate of recurrent MI arising from non-culprit lesions and more complete recovery of left ventricular function with less hemodynamic instability and fewer arrhythmias in the early stage after myocardial infarction.

The CR might be safe in hemodynamically stable patients with STEMI and MVD undergoing primary PCI. This meta -analysis showed that CR was not associated with an increased risk of stroke, major bleeding, and contrast-induced nephropathy.

The present meta-analysis has several limitations. The included studies with variability in inclusion/exclusion criteria, endpoint definitions, timing of staged intervention and follow-up time. In addition, publication bias is an inherent limitation of meta-analyses. Finally, few endpoints occurred and it is likely that the analyses are underpowered for individual outcomes. The ongoing COMPLETE (Complete vs Culprit-Only Revascularization to Treat Multi-vessel Disease After Primary PCI for STEMI) and FULL REVASC (FFR-Guidance for Complete Non-Culprit Revascularization) trials will provide important data on the optimal strategy for patients with STEMI and MVD.

## Conclusions

For patients with STEMI and multivessel disease undergoing primary PCI, complete revascularization did not decrease the risk of all-cause mortality in current evidence from randomized trials. However, it is associated with significant reductions in MACE and cardiac death along with a reduced need for repeat revascularization. Immediate complete revascularization might be feasible in STEMI and multivessel disease patients undergoing primary PCI. More studies are needed to confirm the indications for and timing of non-infarct artery PCI.

## Additional files

Additional file 1: **Figure S1** Relative risk for stoke for complete revascularization (CR) versus infarct-related coronary artery (IRA) only revascularization. (JPG 1347 kb)

Additional file 2: **Figure S2** Relative risk for contrast-induced nephropathy (CIN) for complete revascularization (CR) versus infarct-related coronary artery (IRA) only revascularization. (JPG 198 kb)

Additional file 3: **Figure S3** Relative risk for major bleeding for complete revascularization (CR) versus infarct-related coronary artery (IRA) only revascularization. (JPG 1274 kb)

Additional file 4: **Figure S4** Relative risk for all-cause mortality for complete revascularization (CR) versus infarct-related coronary artery (IRA) only revascularization in subgroup analysis of fractional flow reserve (FFR). (JPG 1438 kb)

Additional file 5: **Figure S5** Relative risk for all-cause mortality for complete revascularization (CR) versus infarct-related coronary artery (IRA) only revascularization in subgroup analysis of follow-up time. (JPG 456 kb)

Additional file 6: **Figure S6** Publication bias assessed by funnel plot for all-cause mortality. Squares represent the trails included. (JPG 884 kb)

## Abbreviations

CI: Confidence interval
CIN: Contrast-induced nephropathy
CR: Complete revascularization
FFR: Fractional flow reserve
ICR: Immediate complete revascularization
IRA: Infarct-related coronary artery
IVUS: Intravascular ultrasound
MACE: Major adverse cardiac events
MVD: Multivessel disease
PCI: Percutaneous coronary intervention
RCTs: Randomized controlled trials
RR: Risk ratios
SCR: Staged complete revascularization
STEMI: ST-elevation myocardial infarction
