# Supplementary figures and images for: Complete versus culprit-only revascularization in patients with ST-segment elevation myocardial infarction and multivessel disease: a meta-analysis of randomized trials
Source: BMC Cardiovasc Disord. 2019 Apr 22;19:91. doi: 10.1186/s12872-019-1073-8 (PMC6477715; doi:10.1186/s12872-019-1073-8)

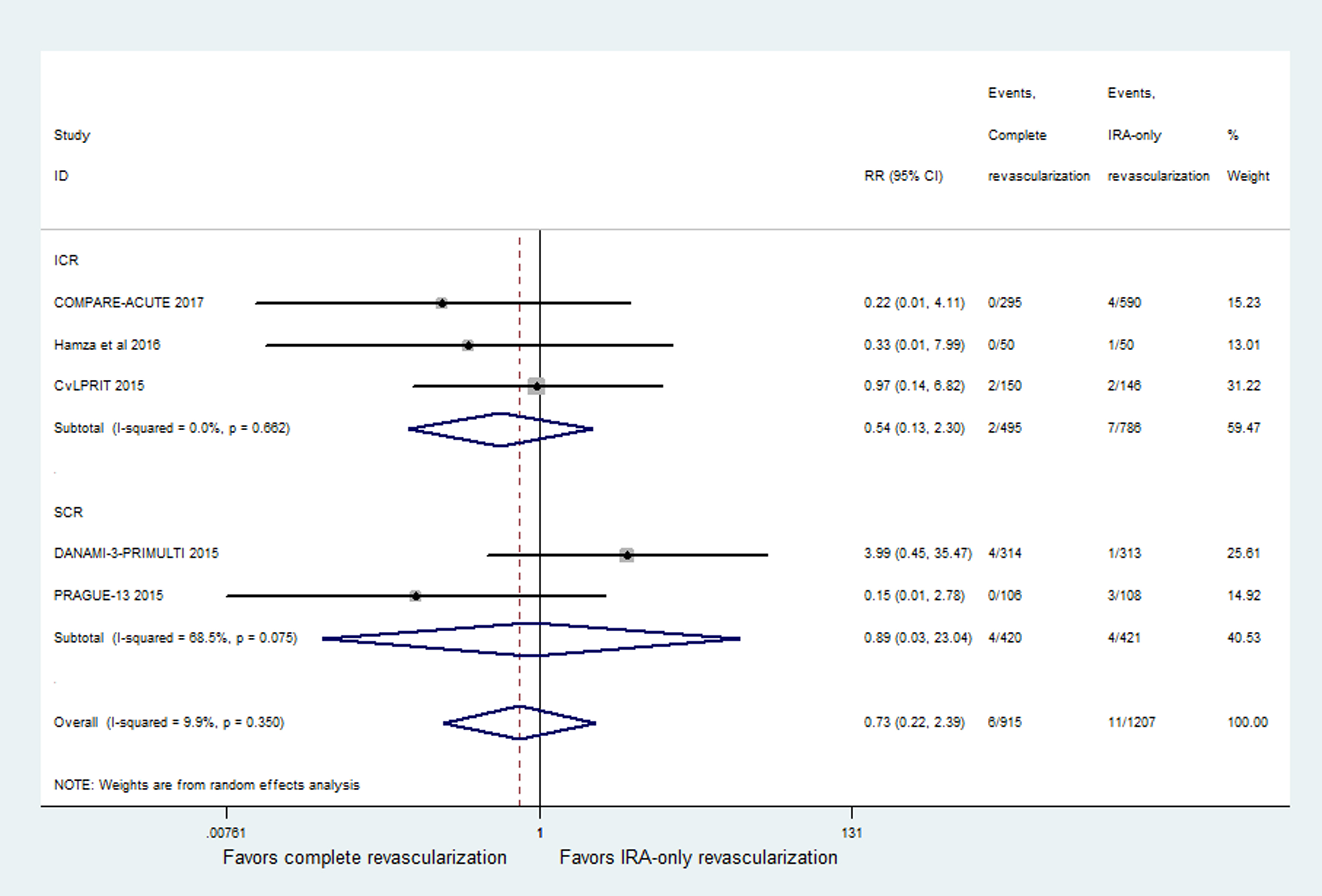

Supplement: Supplementary file 1 — Figure S1 Relative risk for stoke for complete revascularization (CR) versus infarct-related coronary artery (IRA) only revascularization. (JPG 1347 kb) [file 12872_2019_1073_MOESM1_ESM.jpg]

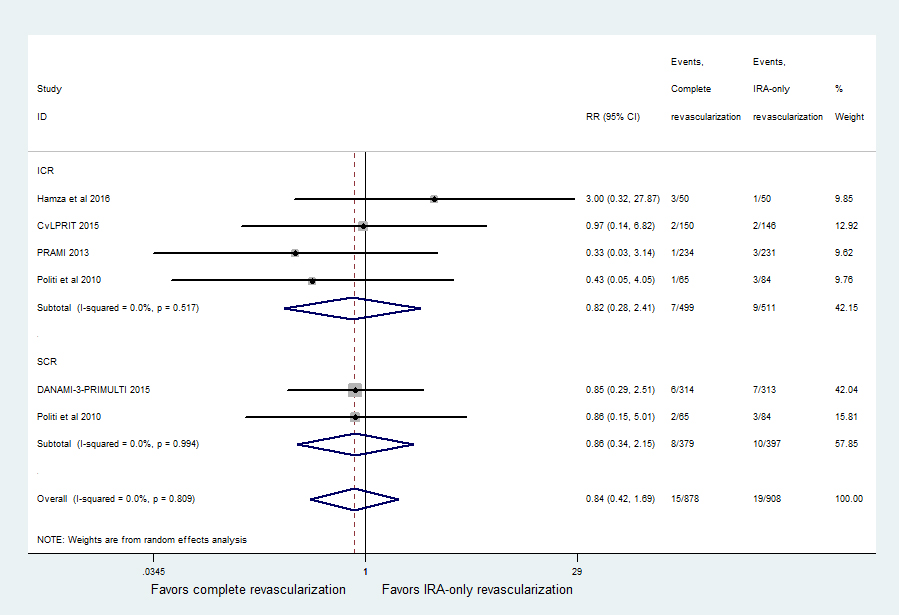

Supplement: Supplementary file 2 — Figure S2 Relative risk for contrast-induced nephropathy (CIN) for complete revascularization (CR) versus infarct-related coronary artery (IRA) only revascularization. (JPG 198 kb) [file 12872_2019_1073_MOESM2_ESM.jpg]

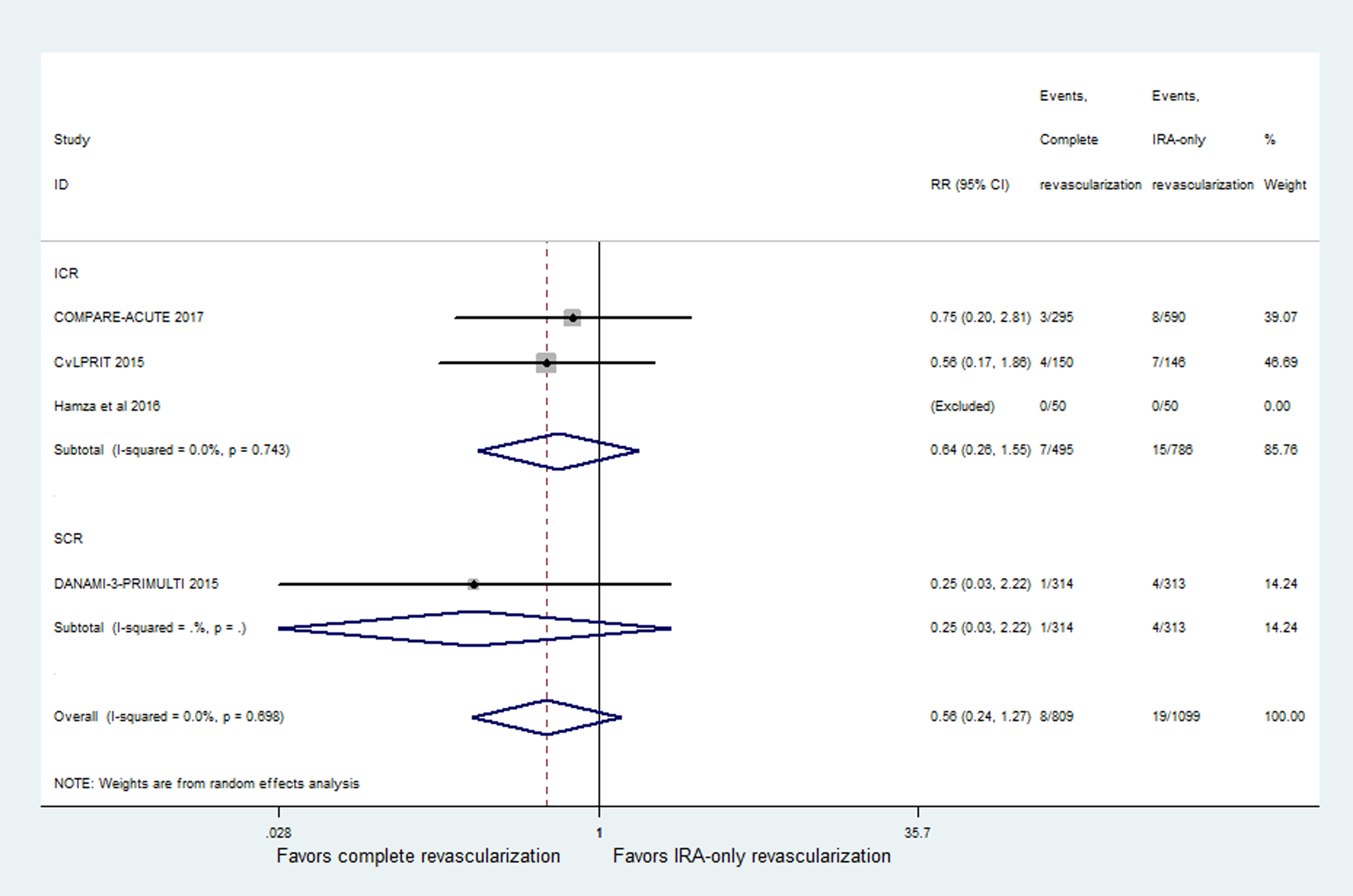

Supplement: Supplementary file 3 — Figure S3 Relative risk for major bleeding for complete revascularization (CR) versus infarct-related coronary artery (IRA) only revascularization. (JPG 1274 kb) [file 12872_2019_1073_MOESM3_ESM.jpg]

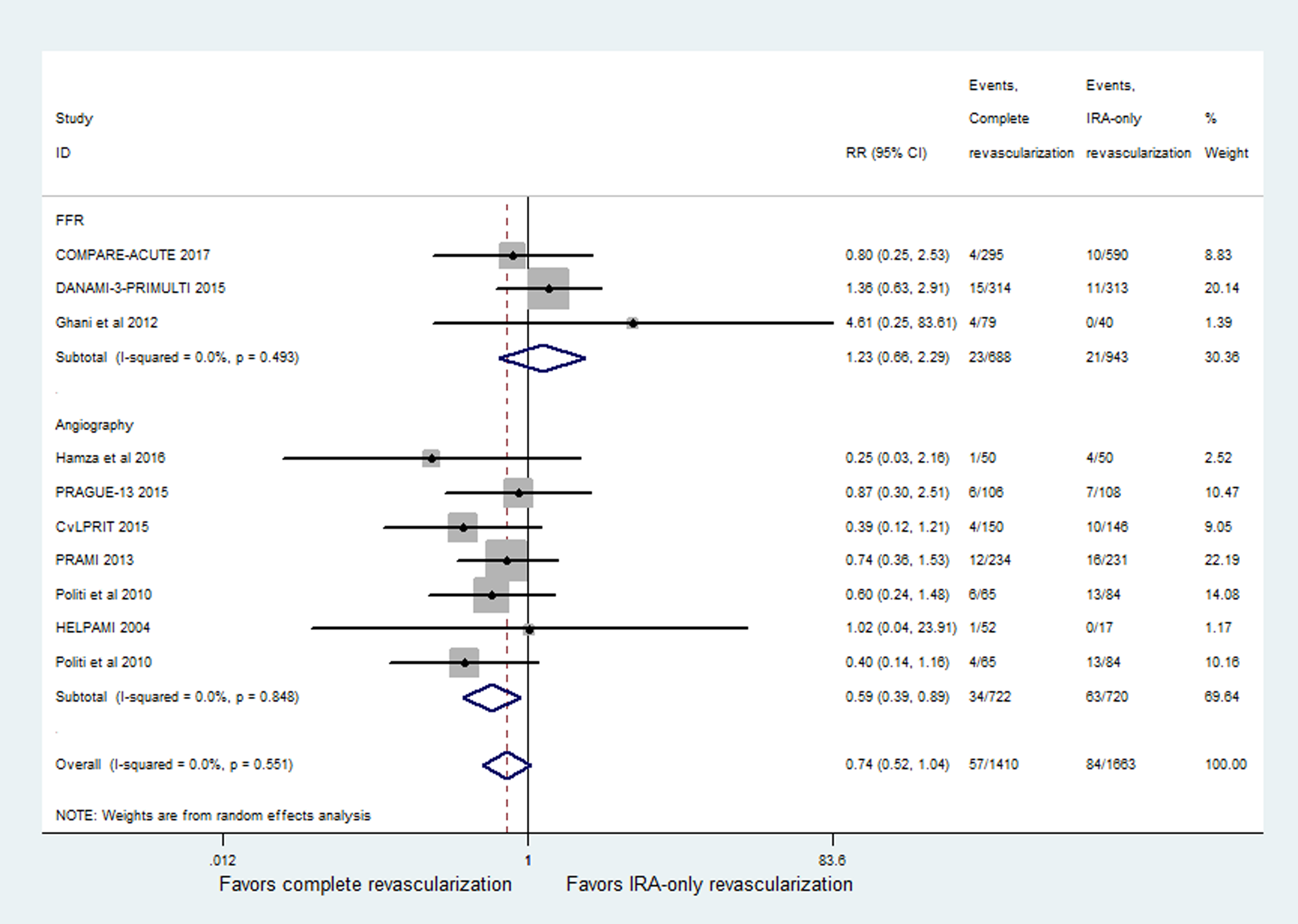

Supplement: Supplementary file 4 — Figure S4 Relative risk for all-cause mortality for complete revascularization (CR) versus infarct-related coronary artery (IRA) only revascularization in subgroup analysis of fractional flow reserve (FFR). (JPG 1438 kb) [file 12872_2019_1073_MOESM4_ESM.jpg]

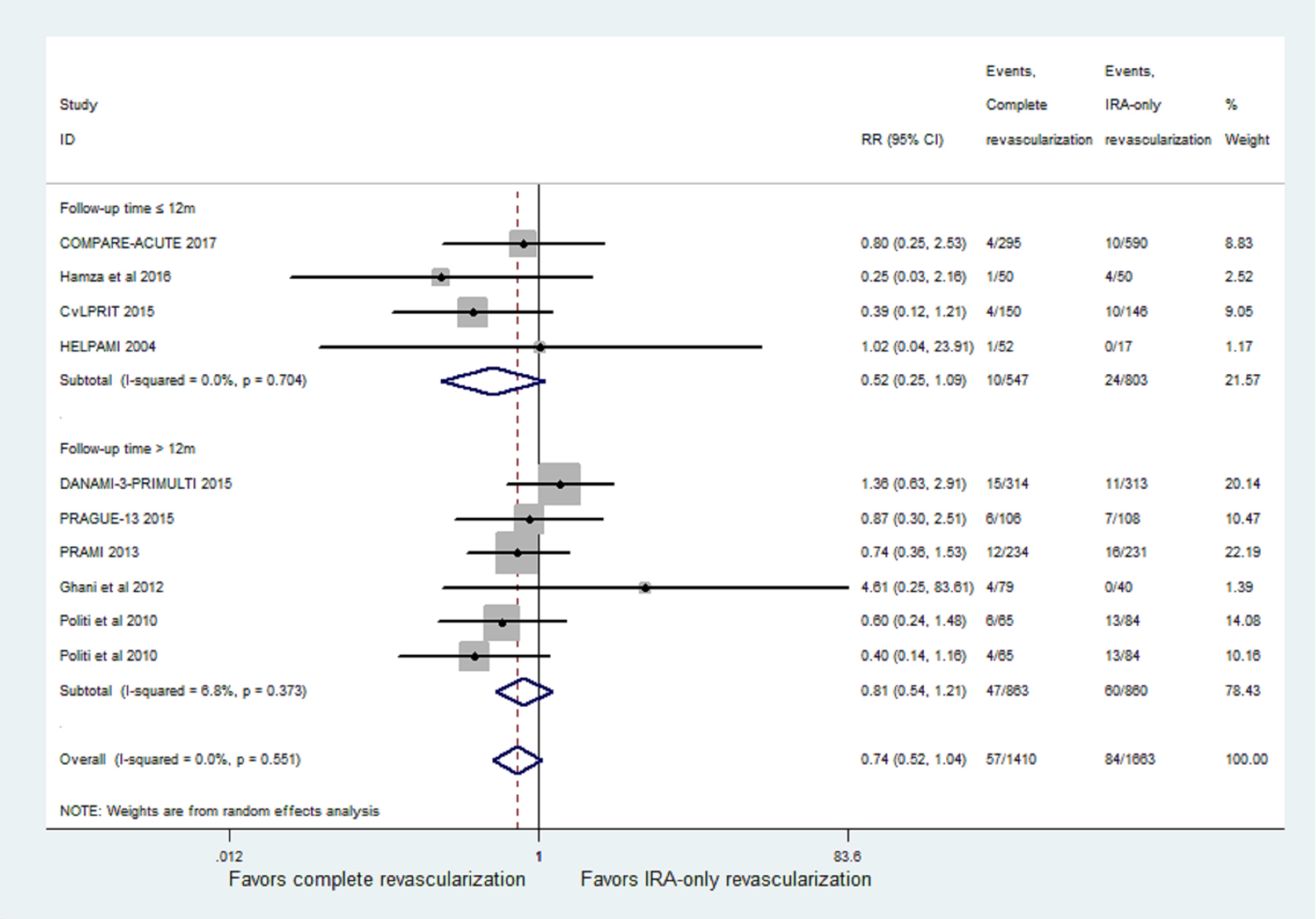

Supplement: Supplementary file 5 — Figure S5 Relative risk for all-cause mortality for complete revascularization (CR) versus infarct-related coronary artery (IRA) only revascularization in subgroup analysis of follow-up time. (JPG 456 kb) [file 12872_2019_1073_MOESM5_ESM.jpg]

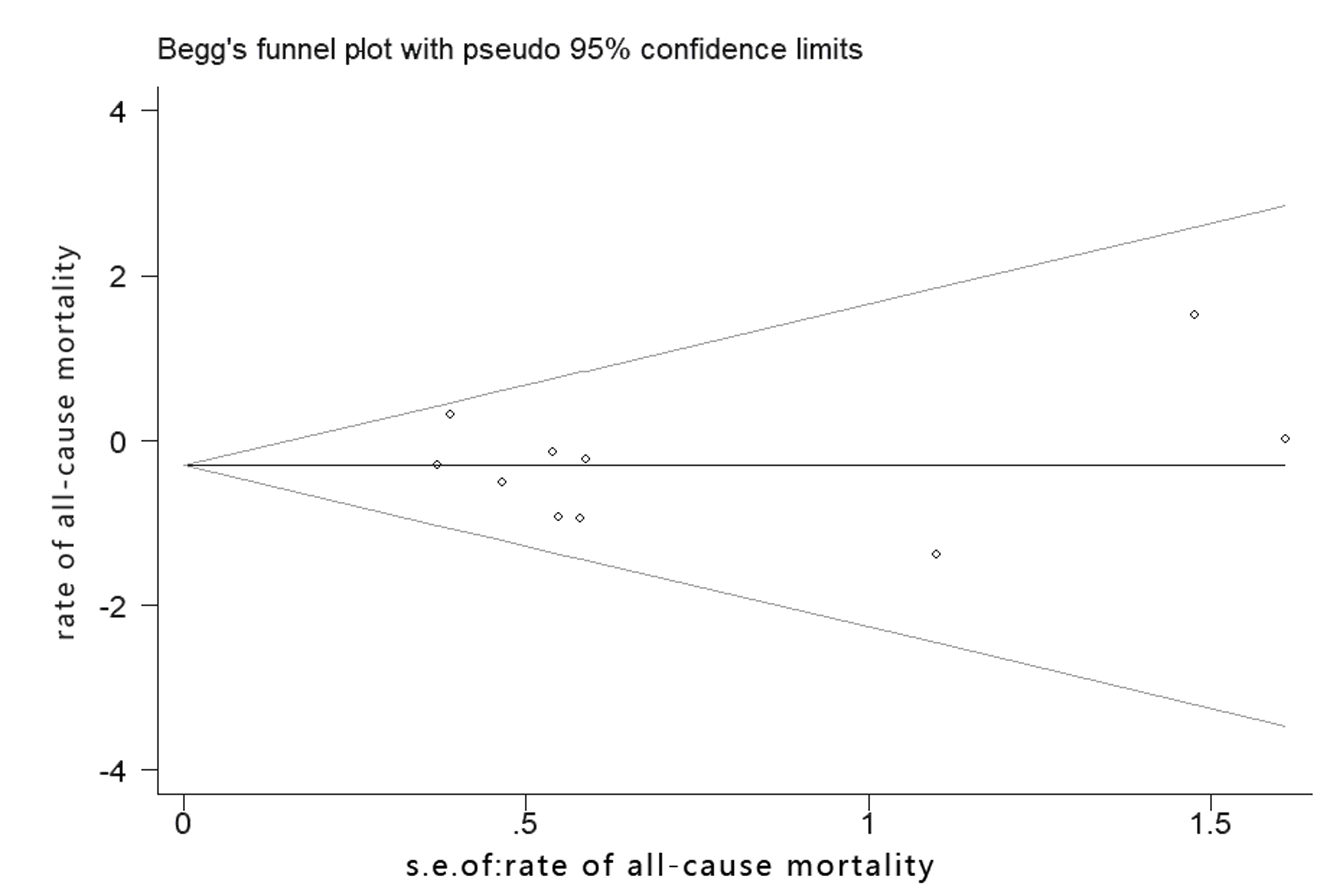

Supplement: Supplementary file 6 — Figure S6 Publication bias assessed by funnel plot for all-cause mortality. Squares represent the trails included. (JPG 884 kb) [file 12872_2019_1073_MOESM6_ESM.jpg]
